# Hospital and Institutional News

**Published:** 1912-09-07

**Authors:** 


### HOSPITAL AND INSTITUTIONAL NEWS.

### THE DETENTION OF CONSUMPTIVES AT ST. HELENS.

On learning last Wednesday of the "Detention of a Consumptive: First Case under a Local Act," even moderately read people must have been irresistibly reminded of Samuel Butler's classic description of a man's trial on the charge of consumption in the courts of Erewhon. What actually happened at St. Helens earlier this week was the application of the deputy town clerk for an order to remove a tuberculous patient to a hospital. Mr. Turton, the deputy clerk, stated that this was the first application of the kind ever made in the country, and he based it on a section of the St. Helens Corporation Act, 1911, which gave power to the magistrates to grant such an order on the certification of the medical officer of health that a person was tuberculous, in an infectious state, and unprovided with proper precautions. Dr. Buchan, the medical officer, gave evidence of the man's infection, and the Bench made an order for the patient to be detained in hospital for three months, which is the full period under the Act.

### A BRIGHTON DOCTOR'S INDEPENDENCE.

Even the layman can hardly fail to note with some interest the way in which obedience to the British Medical Association and disregard of its requests now receive encouragement and now a check, as resignations from the Advisory Committee are announced or withheld. In the process the Association does not fail to lose a member or two. Thus Dr. Duncan Forbes, Medical Officer of Health for Brighton, has resigned from his local branch. Local feeling has been aroused by the following circumstances and suppositions. The Brighton Education Committee advertised recently for a medical officer of inspection, a position which had been treated to a warning notice in the Association's journal. It has been since alleged that Dr. Forbes wrote private letters to medical officers of health all over the country asking them to send candidates for these positions. On this ground the local ethical committee has been considering the question of Dr. Forbes' expulsion from the Association—a course now rendered impossible by his own voluntary resignation. Shortly after we go to press the Brighton Town Council will be asked to appoint Dr. Forbes chief tuberculosis officer under the Insurance Act. If appointed it may be confidently expected that the Association, under the above circumstances, may plead for his resignation in vain.

### MORE INSURANCE INCIDENTS.

Further incidents excited by the Insurance Act have taken place during the past week. The most important is that Mr. Herbert Jones, one of the fourteen medical members of the Advisory Committee which previously signed a resolution stating that for the present it was their duty not to resign, in spite of the British Medical Association's request, has now sent in his resignation. Mr. Herbert Jones is Medical Officer of Health for the Herefordshire combined district. On the other hand, Birmingham, where the British Hospitals Association will hold its annual Conference on the 19th and 20th, has given birth to an Anti-Insurance Act League, which is about to collect signatures for a petition to be sent to the King asking for a dissolution of Parliament, so that the nation may have an opportunity of expressing its opinion on the Act.

### WOMEN RESIDENTS IN POOR-LAW INFIRMARIES.

We publish this week an instructive article on this still controverted subject by Miss Catherine Kirk, M.B., resident assistant medical officer to the Halifax Poor-Law Hospital. As will be seen from her concluding paragraphs, the Halifax Guardians have had a relatively long practical experience of women in resident posts in their infirmary, and show no signs of being dissatisfied with the result. The Halifax Infirmary is an institution with 400 beds, and so women residents cannot escape a wide experience of all sorts of cases, none of which, says Miss Kirk, have been found undesirable for women to deal with. We trust that her arguments and experience may prove useful to the Dewsbury Board of Guardians, who are not impressed with the Local Government Board's statement that their proposal to appoint a woman resident doctor is inexpedient. Mr. George Blacker, who presided at a recent meeting of the Guardians, and Mr. E. T. Tunnicliffe, the deputy clerk, should welcome Miss Kirk's information, for it is likely to throw a clear light on the inquiries they are making at the moment on this subject.

### A GREAT POST-GRADUATE TEACHER.

By the death on September 2 of Mr. Leonard Arthur Bidwell the cause of post-graduate medical education in London loses one who has been conspicuously identified with the movement for some years. His talent for organisation found full scope at the Post-Graduate College attached to the West London Hospital, of which he was for several years the dean. Indeed, it is not going too far to say that his share in the success of the College has been greater than that of any other two men who have contributed to its progress. Not only was Mr. Bidwell a splendid organiser and business manager of this Post-Graduate College, he was also one of its most successful teachers. His classes in abdominal surgery drew students from far and near, and he eventually published a handbook of intestinal surgery largely founded on his experience as a teacher. He also published a volume on "Minor Surgery" a year or so ago which had great merits, though it was not perhaps the equal of his previous composition. Mr. Bidwell, who was only forty-seven, was educated at St. Thomas's Hospital; but his subsequent career has been wrapped up in the West London Hospital, to which he was surgeon. He took a great interest in the West

London Medico-Chirurgical Society, of which he had been president; and had filled a similar position in the Chelsea Clinical Society. In surgical practice his success was also pronounced, especially in those departments of abdominal surgery which he taught so admirably; and both among his patients and his professional colleagues his kindly disposition and genial hospitality brought him troops of friends. His death, moreover, draws attention to the extraordinary havoc made by death and by resignations at the West London in the last few years. A list of the hospital staff at the end of 1908—less than four years ago—suffices to prove this point. Of the physicians to the hospital at that date, only Dr. Beddard remains; Dr. Donald Hood and Dr. Seymour Taylor having been transferred to the consulting staff. Of the surgeons, none remain; Mr. Keetley and Mr. Bidwell have died, and Mr. Edwards has gone to the consulting staff. Of the then assistant physicians, Dr. Davis has resigned in order to devote all his time to the throat and nose department. The assistant surgeons of 1908 are still in the service of the hospital, but there has been a resignation among those appointed to fill vacancies—namely, that of Mr. Etherington Smith. Also Dr. Mansell Moullin is no longer senior gynæcologist; Dr. Drummond Robinson has succeeded to that position, and Dr. Simson has taken his place as assistant in that department. A truly astonishing list of changes in so short a time!

### THE NEW QUARTERS OF ST. PANCRAS DISPENSARY.

The approaching removal of St. Pancras Dispensary from its old quarters in Euston Road to larger premises in Oakley Square deserves notice. A ground-floor and three storeys provide respectively for waiting and consulting rooms; for tuberculosis patients; for the board-room and the quarters of the resident staff. The committee of management will have representatives of the local authority, the local Insurance Committee, and the British Medical Association. In an appeal for £10,000 as an endowment fund the committee urges that "the Borough must have an institution in respect of which it can claim part of the Government grant," and at present the cost of maintenance is expected to be £1,000 per annum. Standing at a corner the new building has an entrance door from either street. Home visitation will play an increasingly important part in the dispensary's activities.

### THE REPORT ON THE DUBLIN HOSPITALS.

The Board of Superintendence of the Dublin Hospitals has issued its annual report. Nine hospitals, including general, special, and the House of Industry hospitals, have received grants from Parliament. In the course of their inspection the Board "made inquiries respecting the current expenses of each hospital, and the system of purchasing, storing, cooking, and issuing supplies," and the results of their observations are to be found in an appendix where two sets of tables furnish an account of the work done and the cost at which it is done respectively. It will occur to many that the information contained in these tables is not made as fruitful as it might be; that the records are there, but not the deductions. Yet such a report as this should form a basis on which many of the old debated questions—a central board for purchasing supplies and what not—might be discussed. For instance, we are always warned that when the highest and lowest prices paid by different hospitals for the same article are compared it must not be thought that the lowest price was a possible one for every institution. That is obvious, but cannot some method be devised by which the individual conditions which invariably produce such varied figures can be harmonised so that a fair average may be collectively struck? Experience proves that a little deduction causes a closer study of figures than anything else.

### THE BRADFORD INFIRMARY SCHEME.

There is, perhaps, little doubt that the Bradford new infirmary scheme has hung fire of late, and, therefore, sincere thanks ought to be paid to the Bradford Co-operative Society for the subscription of £500 which they have voted for the scheme. True, one enterprising member who moved to double this amount found no seconder, but that does not detract from the substantial value, and the consequent advertisement of its object which arises from the gift. The President of the Co-operative Society, Mr. F. Denman, moved the resolution, which empowered the Royal Infirmary to allocate the sum at its discretion, that is to say, either to the endowment or to the building fund. Now that the holiday season is drawing to a close with the advance of September an opportunity arises for once more attracting attention to the scheme. There is much experience to prove that the autumn is one of the best periods for the issue of charitable appeals, and we hope that those responsible for the Bradford Infirmary scheme will not forget to act on this when October comes round.

### SWANSEA HOSPITAL AND THE INSURANCE ACT.

Dr. W. L. Griffiths, honorary secretary to the honorary medical staff of the Swansea Hospital, has addressed a letter to the Board of Management which states that every member of the honorary medical and the consulting staff, with one exception, has signed the following pledge of the British Medical Association: "After that portion of the National Insurance Act referring to medical benefit comes into operation, and until the terms and conditions of administering medical benefit under the National Insurance Act have been approved by the profession, I will not, except in cases of urgent necessity, render professional service to an insured person through the service of any voluntary medical charity. I will not co-operate with any member of the profession who is under contract to render service to insured persons upon terms which are not approved by the profession."

### THE LEICESTER CHILDREN'S HOSPITAL.

The work of reconstructing the Children's Hospital at Leicester has now been taken in hand, and the site of the new sanitary towers is being cleared by the demolition, now partly completed, of the former nurses' home. Nine months is the time which the entire work is expected to require, and the cost is estimated at £10,000. Of this sum more than one-tenth is at present in hand, but, of course, every gift that is received prior to the completion of the work has what is virtually an increased value.

### PHARMACISTS AND UNFAIR COMPETITION.

Under the heading "Wanted to Purchase," the following advertisement appeared last Saturday in a London daily paper: "Dental Diploma or Certificate (Foreign) wanted; a dental student, apprenticed 7 years to L.D.S., Glas., in the Colonies, wants to know the best and quickest way to obtain above; American preferred; expert operator; age 38. Address to etc., etc." That this kind of thing is possible at all is decidedly harmful to the community. A little legislation, based on equity and with no protection of vested interests, is eminently necessary. A correspondent pursues our topic of last week into the field of economics, criticising the rates of pay now offered to the institutional dispenser, whither other hospital officers may care to follow him.

### THE NEW WOMEN'S HOSPITAL.

The rumours which have long been current to the effect that South London was to have a new hospital to be staffed by women doctors are now confirmed by public announcement. The committee in charge of the scheme includes Miss Davies-Colley, M.D., Miss Chadburn, M.D., the Hon. Mrs. Franklin, and Lady Chance, and a freehold site abutting Clapham Common has been secured, while the out-patient department, to be in charge of a woman almoner, will be a separate building situated on the main thoroughfare, and is expected to be opened in the autumn. There will be both general and private wards in the new hospital. The latter will be provided at an inclusive rate of from one to three guineas a week, for the aim is to provide for that class which cannot afford the fees of the private nursing home and yet can afford to pay something. The cost is expected to be £50,000, of which some £10,000 has been promised, although, of course, no public appeal has yet been made. Thus the Royal Free Hospital, with its women doctors on the honorary staff, and the new Hospital in Euston Road, which has a staff of women, will have a peer in South London. Many hopes will be realised, and the large number of women patients who have to be turned away from the above-mentioned hospitals will soon have another institution to which to apply. Needless to say the scheme has the whole-hearted support of women practitioners, and many prominent medical men are strongly in its favour.

### A TRANSFORMATION AT CHARING CROSS.

Extensive alterations are now being made in the medical school attached to Charing Cross Hospital. So great are the structural changes and adaptations that we fear even fairly recent students would wander around their old school with an acute feeling of strangeness and a loss of the sense of orientation. Under the guidance of the Dean, Dr. Wm. Hunter, hitherto neglected corners and rooms are being transformed into light and airy theatres and laboratories. The capacity of the school seems to have been doubled, and the laboratories when fully equipped will probably justify the boast that they are the finest in London for the study of those subjects connected with sanitary science. Under a new scheme the University of London has transferred the whole of its public health and bacteriological departments from King's College to these laboratories—seven in number—in Chandos Street, Strand, and with them the professorial and teaching staffs. So that now in a few weeks the London University laboratories in public health will be established and adequately housed in the buildings of the Charing Cross Hospital Medical School. A view of the accommodation provided and a glance at the names of the teaching staff augur well for the development of this scheme for the teaching of those subjects which are now of such increasing importance in modern medicine. We have had occasion before to refer to the enlargement of the pathological laboratories in this school, for which Dr. Wm. Hunter was largely responsible, and which have developed the teaching in this subject to an extent highly creditable to the school and the special teachers. This further new development cannot fail to enhance the reputation of this medical college.

### HOSPITAL BUILDING IN DUNDEE.

An important extension to the King's Cross Hospital, Dundee, is to be undertaken at a cost of £11,833. A series of blocks is to be added, which will include a measles pavilion. Discharge, kitchen, and administrative sections are also to be built. Mr. James Thomson, the city engineer, pointed out that if cheaper forms of construction were employed numerous modifications would result, although the cost of upkeep would be slightly more expensive. Dr. Templeman, however, spoke strongly against wooden walls, floors or stairs, especially in a pavilion for children, and the result was that the original plans were decided on. The Chairman, Mr. P. M'Cabe, was strongly in favour of this course. We may note in passing that of the sum total the most expensive item was the measles pavilion, which will contain forty beds and cost £5,293. The administrative block comes next in order at some £1,000 less, while the cheapest item is the discharge block. There is, no doubt, a growing reaction against the tendency, which has been well marked in Germany till very lately, to spend large sums in an endeavour to make the administrative block imposing. The portion of a building on which to economise is undoubtedly this, and how graceful simplicity can be is well seen in the new surgical block that has been built at Bristol, which was described in a special illustrated interview in these columns a few weeks ago.

### A NEW APPOINTMENT SUGGESTED.

The growing public organisation of the different branches of our medical service, of which just now the anti-phthisis campaign is the most prominent, has done more than anything else to increase the number of appointments of late years. As an outcome of the notification of consumption as an infectious disease comes this latest suggestion from the Secretary of the National Medical Union, that "a whole-time tuberculosis officer" should be appointed to assist the already overworked medical officer of health and "to act as consultant in friendly co-operation with the local family practitioner." The scope and duties of the proposed new post are but vaguely defined, and the feasibility of separating the duties of tuberculosis officers from those of the medical officers of health can only be tested when the present voluntary and State efforts to combat consumption have become more defined and correlated than can be expected for a few months. Medical officers of health are likely to be in two minds about Mr. J. Webster Watts' proposal.

### A SURVIVOR OF THE MUTINY.

Another Mutiny veteran has passed away with the death of Surgeon-Major Bernard Kendall at the age of eighty-one. In the early fifties he was assistant surgeon in the Honourable East India Company, and later held a similar post at the General Hospital at Calcutta. When the Mutiny broke out in 1857, he was stationed some fifty miles from Lucknow with an infantry regiment at Secrora. On the mutiny of this regiment he escaped with some brother officers and Sir Charles Wingfield, his wife having previously taken refuge in Lucknow. Major Kendall retired after twenty-one years in the Indian Medical Service.

### BUILDING AT £55 PER BED.

In order to prevent accident cases, which are numerous in the local collieries, from having to be removed to Newcastle Infirmary, it is again proposed to build a hospital of eighteen beds and six cots at Ashington. The local nursing association has more than once endeavoured to push forward this scheme, and at a recent meeting explained through its secretary that it was prepared to erect "a plain, substantial hospital for twelve men, six women, and six children." "It was anticipated," said Mr. Craigs, "that a capital expenditure of £1,000 would suffice for the erection and equipment of such an institution." That is to say, the cost was anticipated to work out at rather less than £55 per bed. An optimistic estimate, to say the least, and many a committee would be glad to hear of the architects and the designs that promised such a result combined with efficiency. Mr. W. H. Hughes tried to correct this rosy impression by referring to an institution with which he had been connected, where the cost per bed had proved to be about £400. We fear this is a commoner experience, but record the lower figure as one which architects would do well to aim at in future!

### AN IMPORTANT COTTAGE HOSPITAL EXTENSION.

The Cirencester Cottage Hospital, which has often been cited as a good example by those whose business it is to understand the progress of the voluntary system, is to increase its powers of accommodation. The proposed plans, which will raise the number of beds from eleven to seventeen, have been the subject of anxious care to the committee and to Mr. V. A. Lawson, who has been consulted and has brought to the task a careful comparison of existing hospitals. The cost is to be borne by Mr. D. G. Bingham, who is announced to have undertaken to defray all expenses connected with the extension. His generosity is to be recorded by the christening of the new ward after him. The work is to be begun at once, and the cost is expected to be about £2,000.

### A NEW INFIRMARY FOR DUDLEY.

Permanent provision for the proper treatment of in- and out-patient eye cases is to be made at Dudley, Worcestershire, by the erection of a new eye infirmary. The new institution will be built on a site which adjoins the existing hospital. It will have accommodation for eighteen in-patients and a theatre. The present surgical out-patient department will be replaced by a much more elaborate building with full equipment for all out-patient cases. The present in-patient department will continue its work, but after the 30th of this month the out-patients will be attended to in temporary quarters in a separate building. The building scheme has been under consideration for two years. It is seven years since an eye department was established in connection with the Dudley Guest Hospital and the Dudley Dispensary. But even at that time it was realised that this arrangement could be only a temporary one.

### THIS WEEK'S DRUG MARKET.

Some improvement in business in the Drug market is noticeable, and there is an advancing tendency in the prices of several articles. Tartaric acid, citric acid, and cream of tartar are in fair demand, and there is a possibility of an advance in the value of the two acids. Balsam tolu is again dearer owing to scarcity, and cascarilla is likely to advance in price owing to the same cause. Buchu leaves are dearer, and still higher quotations are expected. A good business has been done in eucalyptus oil, which is likely to become scarce and dearer during the coming winter; it would probably be wise to make purchases at an early date to cover requirements for the next few months. Sandalwood oil is dearer. Another advance in the price of santonin is expected. Cardamons are dearer, and the price is likely to advance still further. Owing to recent bad weather in Belgium quotations for chamomiles are on a higher scale. An upward movement in the price of quicksilver has been announced, and mercurials are proportionately dearer. The unexpected advance in the price of opium is well maintained. Cod-liver oil is dearer, and as the principal consuming season is approaching a decline in price is hardly expected.

### TO OUR READERS.

Contributions are specially invited from any of our readers to these columns. They should deal with topical subjects and news. They must be authenticated for the information of the Editor only. The minimum payment if published is 5s. There is no hard-and-fast rule as to space, but notes of about twenty lines in length are preferred.

